# *In ovo* injection corticosterone method for physiological and behavioral studies in chickens

**DOI:** 10.1016/j.mex.2020.100908

**Published:** 2020-05-11

**Authors:** Abdelkareem A. Ahmed, Mohammed Elmujtba Adam Essa

**Affiliations:** a Department of physiology and Biochemistry, University of Nyala, Nyala, Sudan; b Institute of Molecular Biology, University of Nyala, Nyala, Sudan; c Department of One Health, Medical and Cancer Research Institute, Nyala, South Darfur State, Sudan; d Biomedical Research Institute, Darfur University College, South Darfur State, Sudan

**Keywords:** In ovo injection, Corticosterone, Chicken egg, Chicks, Aggressive behaviors

## Abstract

The phenotype of organisms is not only influenced by genetic factors, but also by environmental factors that play a critical role in shaping their morphology, physiology, behavior and reproductive capacity. In avian species, maternal influences have aroused much attention after the discovery that avian eggs contain a variety of maternal derived steroid hormones. Precocial birds offer a useful animal model so as to solve the mother-offspring interference problem. By removing the maternal effect, scientists can evaluate the effect of glucocorticoid exposure during the embryonic development and its effects on later of phenotypic traits. However, the study of bird's aggressive behaviors using in ovo injection of hormone has not been reported. We used in ovo injection of corticosterone to study aggressive and fearfulness behaviors in chicken in their life later.

• Fertilized chicken egg consider as pregnant mother
• In ovo injection of corticosterone by pass mother-offspring interference problem
• The method allow scientist to evaluate the influences of stress hormone on embryonic development and its later life consequences

**Table:** Specifications Table

Subject Area:	*Select one of the following subject areas:*
	* • Biochemistry, Genetics and Molecular Biology:*
	* • Veterinary Science and Veterinary Medicine*
More specific subject area:	*Describe narrower subject area*
	*Physiology and Behavior*
Method name:	*In ovo injection of corticosterone*
Name and reference of original method:	[2,3]
Resource availability:	*If applicable, include links to resources necessary to reproduce the method (e.g. data, software, hardware, reagent)*

## Method details

**Fig. 1:**
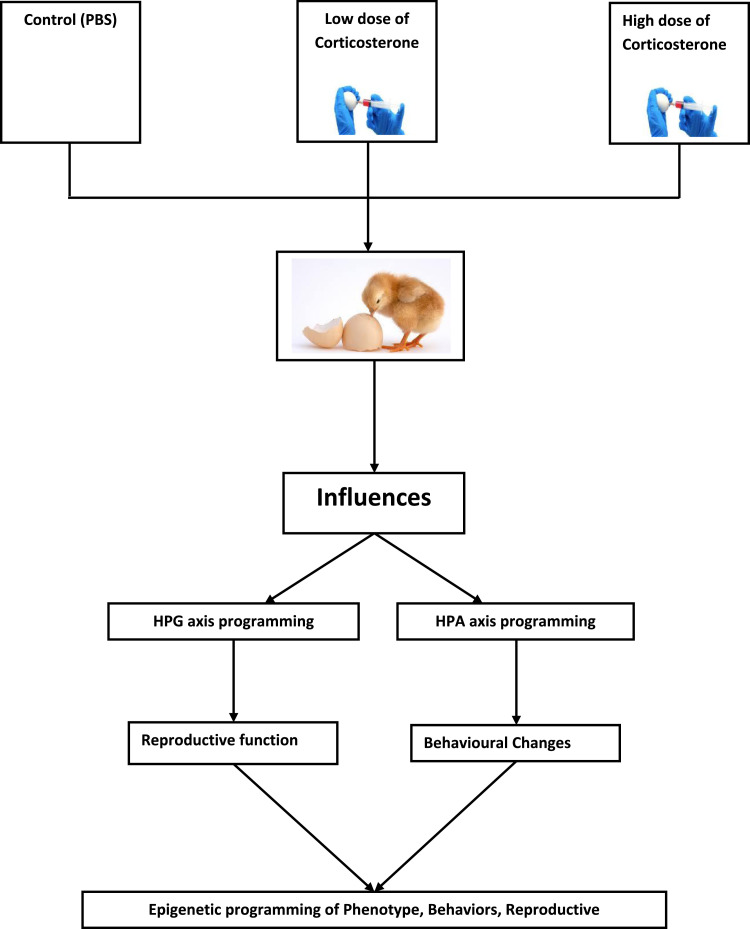

In this method, we used fertilized eggs from eggs laid by hens one month after onset of lay and randomly divided into three groups (Fig. 1). Corticosterone (CORT) was dissolved in absolute alcohol, rather than the oil which we found in our previous experimental trials that affect chicken embryonic development, and diluted in phosphate buffer saline (PBS) to prepare doses that were required for in ovo injection for behavioral observations. The doses of CORT used were 0.2 µg as low doe and 1 µg as high does completed with a volume of 100 µL solution containing a minimal amount of alcohol. The high and low CORT dose was determined according to previous publications [2,3] that used to study stress response, oxidative stress and telomere length in juvenile. In this method, we considered the CORT levels that we measured in our earlier publication which was: yolk (3-4 ng/g) and the albumen (0.5 ng/g) [1] to decide low and high doses. Prior to egg incubation, the eggs were firs washed with alcohol as aseptic procedure, and then injected with PBS as control, 0.2 µg and a 1 µg dose of CORT under aseptic conditions. Eggs were injected randomly by advancing a Hamilton syringe into a hole in the middle of the long axis until the yolk membrane was penetrated (approximately 20 mm below the surface). The incubation conditions were set according to our previous experiment [6]. In second experiment, we incubated the eggs for 10 days, and then the eggs were put out and injected CORT in ovo at the same doses and then eggs were again incubated. All eggs were incubated in a forced draught incubator with automatic turning every two hours at 37.5 ± 0.3 C and 55% to 57% humidity. In both experiments, chicks were hatched inside the incubator and were left to dry completely (approximately 12 h) before chicks were removed. The hatchability of the eggs ranged from70% to 75% and no clear differences in hatchability or hatching time were observed within groups and among two experiments. One-day-old chicks were individually weighed, wing labeled, and put into battery cages with constant fluorescent lighting condition. During the first week, the room temperature was adjusted to 32–35°C, and then decreased gradually to 3°C per week until 21°C. Both sexes were transferred to floor pens covered with sawdust litter. The stock density was 20–25 kg/m^2^. The relative humidity was maintained at 40–60%, and the lighting, ventilation, as well as the feeding and management procedures complied with the Feeding Management Regulations of Yellow-feathered Chicken (NY/T 1871-2010).

**Fig. 2:**
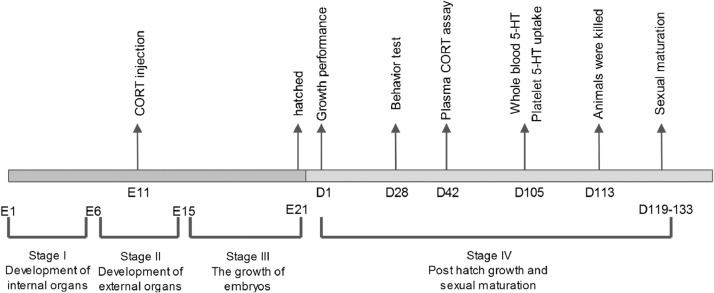

For the first experiment, growth performance, behavior test, plasma CORT measurements, measurement of whole blood 5-HT and platelet 5-HT uptake we measured as shown in (Fig. 2)

**Fig. 3:**
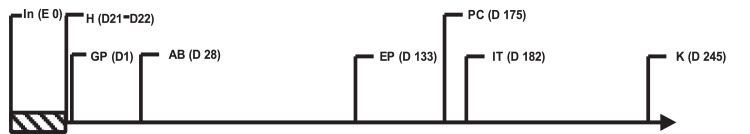

For second experiment, growth performance, egg production, plasma CORT, Behaviors test and tonic immobility tests were measured as shown in (Fig. 3). For both experiments, we used 6 chickens per group, in total of 18 chickens for all experimental parameters except the growth rate that we used all chickens. To minimize the stress caused by different manipulations, we used different batch of 6 birds for different measurements.

## Aggressive behavior test

The behavior tests were performed as described previously [4]. Briefly, 30 chickens from each group which were unfamiliar to each other from different brooders were placed in an experimental arena (similar in size and structure to their brooders where chickens have been raised) which was established in a room familiar to the animals. The room was visually and acoustically isolated from the aviary. For visual identification, chickens were marked with different colors (red, green, blue) on different locations (head, back and tail). Neither the colors nor their locations affected the behaviors of chickens in the present study. The chicken's behavior was videotaped during a 60 min period. The number of aggressive attacks of each individual was recorded, and aggression was defined as a chicken pecking, grabbing, twisting skin on the head and nape of the other chicken. The observer who recorded and analyzed the aggressive behaviour was not aware of the experimental treatments.

## Tonic immobility (TI) test

TI tests were measured using different chickens. The TI tests were measured according to the method described previously [5]. Briefly, a chicken was carried individually to another isolated room devoid of other birds. The chicken was placed on its back on the floor and restrained for at least 20 s (with one hand on the sternum and one lightly cupping the head of the bird). The experimenter remained silent and virtually motionless in the room, out of the bird's sight. The TI duration was considered between 10 and 600 s. If the chicken terminated in **<**10 s, it was captured, and the trial was repeated. If TI was not attained after 3 attempts, a score of 0 s was given. Conversely, if the bird failed to right itself after 10 min, the test was terminated and a maximum score of 600 s was given for tonic immobility duration.

## Declaration of Competing Interest

The authors declare that there is no conflict of interests.
